# Plasma Metabolites of One-Carbon Metabolism Are Associated With Esophageal Adenocarcinoma in a Population-Based Study

**DOI:** 10.14309/ctg.0000000000000879

**Published:** 2025-06-26

**Authors:** Shailja C. Shah, Maria Alejandra H. Diaz, Xiangzhu Zhu, Teodoro Bottiglieri, Chang Yu, Lesley A. Anderson, Helen G. Coleman, Martha J. Shrubsole

**Affiliations:** 1 Division of Gastroenterology, University of California, San Diego, La Jolla, California, USA;; 2 Gastroenterology Section, VA San Diego Healthcare System, La Jolla, California, USA;; 3 Vanderbilt University School of Medicine, Nashville, Tennessee, USA;; 4 Division of Epidemiology, Department of Medicine, Vanderbilt Epidemiology Center, Vanderbilt University School of Medicine, Vanderbilt University Medical Center, Nashville, Tennessee, USA;; 5 Center of Metabolomics, Institute of Metabolic Disease, Baylor Scott & White Research Institute, Dallas, Texas, USA;; 6 Division of Biostatistics, Department of Population Health, Grossman School of Medicine, NYU Langone Health, New York, New York, USA;; 7 Aberdeen Centre for Health Data Science, Institute of Applied Health Sciences, School of Medicine, Medical Sciences and Nutrition, University of Aberdeen, Scotland, UK;; 8 School of Medicine, Dentistry and Biomedical Sciences, Center for Public Health, Queen's University Belfast, Belfast, Northern Ireland, UK;; 9 Division of Epidemiology, Department of Medicine, Vanderbilt Epidemiology Center, Vanderbilt University School of Medicine, Vanderbilt-Ingram Cancer Center, Vanderbilt University Medical Center, Nashville, Tennessee, USA.

**Keywords:** methionine, *S*-adenosylmethionine, *S*-adenosylhomocysteine, Barrett's esophagus, esophageal adenocarcinoma

## Abstract

**INTRODUCTION:**

Esophageal adenocarcinoma (EAC) develops through histopathological stages, including Barrett's esophagus (BE). We analyzed the associations between plasma levels of one-carbon metabolism factors and risks of long-segment BE or EAC.

**METHODS:**

Plasma levels were measured from an Irish population-based case-control study (Factors INfluencing the Barrett Adenocarcinoma Relationship study; 204 long-segment BE cases, 211 EAC cases, and 251 controls). A “methyl replete score” was derived by assigning a score of 0 (<median) or 1 (>median) to the levels of 3 dietary methyl donors (methionine, choline, and betaine) and summing across the metabolites. Multinomial logistic regression models were used to estimate odds ratios (ORs) and 95% confidence intervals (CIs) for the associations between EAC or BE and sex-specific quartiles or score using the lowest level as the reference category and adjusted for potential confounders.

**RESULTS:**

Highest methionine, betaine, vitamin B6, and choline levels were all associated with 62%–82% reduced risks of EAC (*P*_trends_ < 0.001). Conversely, S-adenosylmethionine, S-adenosylmethionine/S-adenosylhomocysteine ratio, total homocysteine, and cystathionine were associated with a greater than 2-fold increased EAC risk. A higher methyl replete score was associated with reduced EAC risk (OR 0.33; 95% CI 0.16–0.66). The highest vs lowest plasma methionine levels were borderline statistically significantly associated with long-segment BE (OR 0.55; 95% CI 0.28–1.07), but all other associations were null.

**DISCUSSION:**

Several biomarkers of one-carbon metabolism are associated with EAC risk, particularly markers of dietary methyl group donors. Future studies to replicate and prospectively evaluate these markers are warranted.

## Graphical abstract

**Figure:**
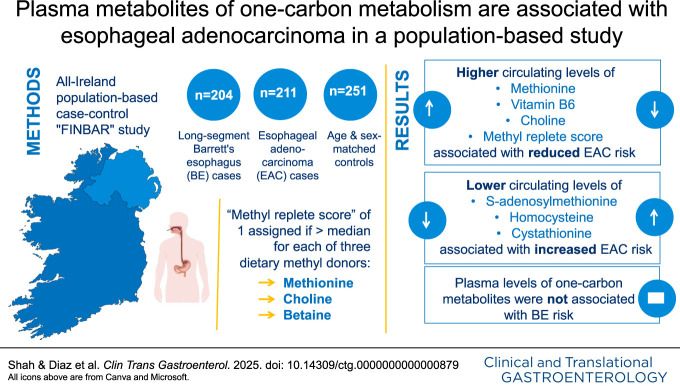

## INTRODUCTION

Esophageal cancer is the eighth most common cancer and the sixth most common cause of cancer-related deaths globally (1). Esophageal adenocarcinoma (EAC) survival remains poor and reflects the fact that most EAC is diagnosed in the advanced stage when symptoms prompt diagnostic workup (1,2). This is in stark contrast to stage I EAC, which can be treated with curative intent.

It is generally accepted that most, if not all, EACs develop as a stepwise progression of histopathological stages, starting with the replacement of normal esophageal mucosa by intestinal-type mucosa—a preneoplastic condition known as Barrett's esophagus (BE)—followed by dysplasia in a small percent of individuals, with even fewer undergoing malignant transformation to EAC (3). Multiple risk factors have been identified for both BE and EAC (4). Longstanding gastroesophageal reflux (GER), which affects up to 30% of the population, is one of the strongest risk factors of BE (5). Not all patients with GER will develop BE, and among those with BE, only a fraction (an estimated 0.2%–0.5% annually, on average) will have malignant transformation (6). Unfortunately, the molecular mechanisms connecting chronic GER to BE development and the subsequent mechanisms driving neoplastic progression to EAC are not yet well defined, thus highlighting a major knowledge gap. Developing a more precise signature to identify patients at high risk of progression to BE, and from BE to EAC, is needed to reduce the burden of endoscopic surveillance and improve EAC outcomes.

Mutations in several genes have been identified in EAC (e.g., *TP53*, *CDKN2A*, *SMAD4*, *PIK3CA*, among others), some with overlap in BE. Despite the high mutational burden, BE has a relatively stable genome and it is the loss of genome stability that leads to neoplastic progression. One theory is that the cumulative buildup of epigenetic modifications—primarily, aberrations in DNA methylation—may ultimately lead to BE with or without subsequent neoplastic progression (7–9). At least 4 subclassifications of EAC are recognized based on specific DNA methylation patterns; these subclassifications have prognostic and therapeutic implications, thus underscoring the importance of DNA methylation in BE and EAC (10,11). Aberrant DNA methylation patterns seem particularly critical in the early stages of neoplastic transformation. S-adenosyl-methionine (SAM) serves as the universal methyl donor that is needed for all methylation reactions, including methylation of nucleic acid (DNA, RNA) and histones. As the sole source of SAM, methionine, an essential amino acid, is a key player in the network of one-carbon metabolism pathways. Methionine itself is also involved in various biological processes that, if dysregulated, have carcinogenic potential. We previously reported that dietary intakes of other elements involved in one-carbon metabolism (folate and vitamin B6) were inversely associated with both BE and EAC risk, suggesting that one-carbon metabolism may be involved in the transformation from BE to EAC, potentially with most relevance early in carcinogenesis (12). These findings were also supported in some previous studies (13–15).

We hypothesized that one-carbon metabolism factors, especially SAM, might be associated with increased risk of BE and EAC. To test this hypothesis, we analyzed the association between plasma levels of one-carbon metabolism factors and risk of nondysplastic BE or EAC using a population-based case-control study of BE and EAC in Ireland, known as the Factors INfluencing the Barrett Adenocarcinoma Relationship (FINBAR) study. To our knowledge, this is the first study to investigate plasma biomarkers of one-carbon metabolism and BE/EAC risk.

## METHODS

### Study population

FINBAR is an all-Ireland population-based case-control study established to investigate the etiology of BE and EAC, and its conception is previously described (16–18). In brief, incident cases of EAC (n = 227), long-segment nondysplastic BE (n = 224), and normal population controls (n = 260) were included between 2002 and 2005. Incident EAC cases were identified from hospital clinical records in the Republic of Ireland or through electronic pathology records in Northern Ireland, and all were histologically confirmed. Patients with endoscopically diagnosed long-segment BE (≥3 cm in length) were recruited if the presence of specialized intestinal metaplasia had been histologically confirmed. Patients with BE with dysplasia, irrespective of grade, were excluded. Population-based controls were adults with no history of BE, esophageal, or other gastrointestinal cancer. Controls were recruited at random in Northern Ireland via through General Practice Master Index and in the Republic of Ireland from 4 General Practices representing urban and rural areas (Dublin and Cork). Participants with long-segment BE (hereafter referred to as BE) and population controls were frequency matched within 5-year age and sex strata to the distribution of subjects with EAC, up to a maximum age of 85 years. Appropriate plasma samples were available from 204 nondysplastic BE cases, 211 EAC cases, and 251 controls, and this subset forms the basis of this analysis.

### Data collection

Trained interviewers collected data including demographics, diet and other lifestyle factors and health history (Table 1). Dietary intake was assessed using a validated 101-item Food Frequency Questionnaire, adapted for the Irish population from a version of the European Prospective Investigation into Cancer and Nutrition Food Frequency Questionnaire (19), by incorporating foods reported as commonly eaten in the North/South Ireland Food Consumption Survey (20). Participants were asked to recall their dietary habits over the 12-month period, 5 years before the interview. Body mass index (BMI), 5 years before the interview, was calculated using self-reported weight (kg) divided by current height (m^2^), as measured by the interviewer. The frequency and duration of GER symptoms, including heartburn, experienced by participants were ascertained. Participants were categorized as having regular GER if they reported GER symptoms at least once weekly or at least 50 times per year 5 years before the interview date (17).

**Table 1.** Baseline characteristics of cases (long-segment BE and EAC) and population controls, FINBAR Study

Characteristics^a^	Controls (n = 251)	BE cases (n = 211)	EAC cases (n = 204)	*P* value^b^
Matching factors				
Age (yr)	63.2 ± 12.7	62.3 ± 11.9	64.7 ± 11.0	0.12
Males	211 (84.1)	175 (82.9)	172 (84.3)	0.91
Other characteristics				
Location				<0.0001
Northern Ireland	119 (47.4)	149 (70.6)	112 (54.9)	
Republic of Ireland	132 (52.6)	62 (29.4)	92 (45.1)	
Smoking status				<0.0001
Never	96 (39.2)	82 (39.1)	41 (20.5)	
Former	105 (42.9)	79 (37.6)	91 (45.5)	
Current	44 (18.0)	49 (23.3)	68 (34.0)	
Occupation type				0.11
Manual	122 (50.0)	121 (58.2)	116 (58.6)	
Nonmanual	122 (50.0)	87 (41.8)	82 (41.4)	
Regular GER symptoms^c^	49 (19.6)	153 (72.5)	99 (48.5)	<0.0001
Regular NSAID use	30 (12.1)	28 (13.3)	22 (10.8)	0.74
*H*. *pylori* seropositive	144 (59.0)	103 (50.7)	92 (48.2)	0.05
Education (yr)	11.9 ± 3.2	11.2 ± 2.9	10.7 ± 2.6	<0.0001
BMI 5 yr prior (kg/m^2^)	27.1 ± 3.9	27.0 ± 4.0	28.5 ± 4.8	0.0002
Alcohol intake				0.04
None	67 (26.7)	56 (26.5)	59 (28.9)	
<Median (20 g/d)	88 (35.1)	95 (45.0)	91 (44.6)	
≥Median	96 (38.3)	60 (28.4)	54 (26.5)	
Alcohol intake (g/d)^d^	26.4 ± 23.3	22.6 ± 25.8	20.9 ± 22.5	0.10
Energy (kcal/d)	2,579 ± 816	2,738 ± 775	2,740 ± 802	0.04
Folate intake (μg/d/1,000 kcal/d)	154 ± 37.0	141 ± 34.9	142 ± 35.4	0.0001
Vitamin B_6_ intake (μg/d/1,000 kcal/d)	1.2 ± 0.3	1.1 ± 0.2	1.00 ± 0.2	<0.0001
Vitamin B_12_ intake (μg/d/1,000 kcal/d)	3.0 ± 0.9	3.1 ± 1.1	3.4 ± 1.5	0.0005

BE, Barrett's esophagus; BMI, body mass index; EAC, esophageal adenocarcinoma; GER, gastroesophageal reflux; NSAID, nonsteroidal anti-inflammatory drug.

a Characteristics presented as mean ± SD for continuous data and n (%) for categorical variables.

b Cases compared with all controls, calculated using the *t* test (continuous variables) or χ^2^ test (categorical variables).

c Heartburn/acid reflux symptoms experienced at least once weekly or >50 times per year >5 years before interview date.

d Among those who drink alcohol.

### Blood sample collection and processing

Nonfasting blood samples were collected on the interview date. EDTA tubes were used for plasma sample collection, which were stored at 4°C (on crushed ice) until centrifugation and processing in the laboratory. These tubes were usually spun within 2 hours of collection, at 3,000 rpm, for 15 minutes, at 4°C. After centrifugation, plasma was pipetted into 2 mL storage tubes (minimum of 0.5 mL to each) and transferred to a freezer (−80°C) for long-term storage. The plasma samples in this study had not undergone any freeze-thaw cycles before analysis.

### Measurement of methionine metabolites

All plasma metabolites were determined by liquid chromatography-electrospray tandem mass spectrometry with stable isotope dilution and multiple reaction monitoring for identification and quantification. Methionine, SAM, S-adenosylhomocysteine (SAH), choline, betaine, and cystathionine were determined as previously described (21,22). Additional liquid chromatography-electrospray tandem mass spectrometry methods were used to determine total homocysteine (tHcy), 5-methyltetrahydrofolate, and vitamin B6 (PLP) in plasma (23–25). For each method, 2 levels of pooled plasma were used as quality controls and were included at the beginning and end of each analytical run. In-house quality control values had to meet a requirement of at least <10% variance for the acceptance criteria. Each laboratory run also included an approximately equal proportion of cases and controls. Thirty-four replicates of a pooled plasma sample were included within and between batches to estimate intrabatch (6.4%–11.9%) and interbatch (0.9%–8.6%) variability as measured by the coefficient of variation.

### Statistical analysis

Sex-specific plasma metabolite levels were categorized into quantiles based on the distribution among controls (see Supplementary Table 1, Supplementary Digital Content 1, http://links.lww.com/CTG/B325). A “methyl replete score” was derived by assigning a score of 0 or 1 to below or above the median, respectively, of the 3 dietary methyl donors (methionine, choline, and betaine) levels and summing across the 3 metabolites. Odds ratios (ORs) and 95% confidence intervals (CIs) were estimated from multinomial logistic regression models adjusted for potential confounders. Factors were considered potential confounders if they were previously shown to be associated with BE or EAC. These included age (26–28), sex (26–28), smoking status (26,29), education (30), BMI (31,32), occupation (17,26,32), alcohol intake (29,33), regular nonsteroidal anti-inflammatory drugs use (18,34), country, energy intake, and select vitamin or mineral supplemental intake (e.g., folate, vitamin B12, vitamin B6) (12). These were adjusted for in the analyses. Sensitivity analyses were conducted to evaluate regular GER symptoms and *Helicobacter pylori* infection status. Stratified analysis was used to evaluate potential effect modification by sex, age, BMI categories, PLP level, *H. pylori* status (35), regular GER symptoms, smoking status, and alcohol intake. Those with missing data for a biomarker were excluded from the applicable analysis.

Two-sided statistical significance was set at an α level of 0.05. All statistical analyses were completed using Statistical Analysis System (SAS Enterprise 7.15).

## RESULTS

Table 1 details the characteristics of the 666 study participants. Most cases of BE and EAC were men, consistent with the demography of EAC and BE. More cases, particularly BE, originated from Northern Ireland compared with the Republic of Ireland. Compared with controls, BE and EAC cases were more likely to have regular GER symptoms, higher intakes of energy and vitamin B12, and lower intakes of folate and PLP. Cases had lower mean intakes of alcohol and had a lower frequency of *H. pylori* seropositivity compared with controls. EAC cases were also more likely to have smoked, have a slightly higher BMI, and have slightly fewer education years than controls. The distribution of the plasma biomarker levels among controls is provided in Supplementary Table 1 (see Supplementary Digital Content 1, http://links.lww.com/CTG/B325).

The associations between biomarker levels and risks of BE and EAC are presented in Table 2. For BE risk, there was no significant linear trend observed with increasing or decreasing levels of SAM, SAH, tHcy, betaine, choline, cystathionine, PLP, or methyltetrahydrofolate. There was a suggestive, but not statistically significant, inverse association between methionine and BE risk (OR 0.55, 95% CI 0.28–1.07, *P*_trend_ = 0.05).

**Table 2.** Associations between one-carbon metabolism biomarkers and risks of long-segment BE or EAC, FINBAR

Plasma biomarker	Controls	Case-control comparisons				
		Single marker model				Multimarker model^a^
		BE cases		EAC cases		EAC cases
	n	n	OR (95% CI)^b^	n	OR (95% CI)^b^	OR (95% CI)^b^
Methionine						
Q1 (low)	63	70	1.00 (ref)	109	1.00 (ref)	1.00 (ref)
Q2	63	71	1.09 (0.61–1.94)	37	0.33 (0.19–0.60)	0.40 (0.20–0.78)
Q3	63	39	0.64 (0.34–1.23)	25	0.26 (0.14–0.50)	0.28 (0.13–0.61)
Q4	62	31	0.55 (0.28–1.07)	33	0.35 (0.19–0.65)	0.27 (0.12–0.59)
*P*_trend_			0.05		<0.0001	0.0001
SAM						
Q1 (low)	63	41	1.00 (ref)	25	1.00 (ref)	1.00 (ref)
Q2	63	67	1.69 (0.89–3.22)	48	2.08 (1.06–4.05)	2.32 (1.04–5.14)
Q3	63	48	0.90 (0.46–1.76)	44	1.55 (0.78–3.10)	1.90 (0.82–4.40)
Q4	62	55	1.29 (0.65–2.56)	87	3.04 (1.56–5.93)	3.75 (1.59–8.82)
*P*_trend_			1.00		0.004	0.002
SAH						
Q1 (low)	63	71	1.00 (ref)	59	1.00 (ref)	—
Q2	62	44	0.60 (0.32–1.13)	48	0.60 (0.32–1.10)	
Q3	63	54	0.77 (0.41–1.45)	41	0.61 (0.32–1.14)	
Q4	62	42	0.74 (0.38–1.45)	56	0.81 (0.43–1.50)	
*P*_trend_			0.52		0.55	
SAM/SAH						
Q1 (low)	63	37	1.00 (ref)	28	1.00 (ref)	—
Q2	62	56	1.30 (0.66–2.56)	43	1.47 (0.74–2.90)	
Q3	63	42	0.97 (0.49–1.96)	56	2.01 (1.02–3.97)	
Q4	62	76	1.57 (0.80–3.06)	77	2.82 (1.46–5.46)	
*P*_trend_			0.28		0.001	
tHcy						
Q1 (low)	63	38	1.00 (ref)	33	1.00 (ref)	1.00 (ref)
Q2	63	70	1.55 (0.81–2.97)	32	0.87 (0.44–1.73)	1.12 (0.51–2.47)
Q3	63	47	0.94 (0.49–1.83)	60	1.45 (0.77–2.72)	1.45 (0.69–3.07)
Q4	62	56	1.09 (0.55–2.14)	79	1.92 (1.02–3.60)	1.40 (0.65–2.99)
*P*_trend_			0.68		0.01	0.17
Betaine						
Q1 (low)	63	69	1.00 (ref)	94	1.00 (ref)	1.00 (ref)
Q2	63	51	0.87 (0.48–1.60)	38	0.37 (0.20–0.66)	0.47 (0.24–0.95)
Q3	63	53	1.04 (0.57–1.91)	36	0.44 (0.24–0.80)	0.74 (0.35–1.59)
Q4	62	38	0.73 (0.39–1.4)	36	0.38 (0.21–0.71)	0.97 (0.42–2.21)
*P*_trend_			0.56		0.001	0.86
Choline						
Q1 (low)	63	60	1.00 (ref)	89	1.00 (ref)	1.00 (ref)
Q2	64	50	0.95 (0.51–1.76)	39	0.42 (0.23–0.76)	0.57 (0.29–1.15)
Q3	63	44	0.79 (0.41–1.5)	40	0.45 (0.25–0.81)	0.43 (0.20–0.90)
Q4	61	57	1.02 (0.54–1.92)	36	0.30 (0.16–0.57)	0.22 (0.10–0.53)
*P*_trend_			0.90		<0.001	0.0002
Cystathionine						
Q1 (low)	64	47	1.00 (ref)	44	1.00 (ref)	1.00 (ref)
Q2	62	59	1.45 (0.77–2.76)	36	0.76 (0.40–1.47)	0.93 (0.43–2.04)
Q3	63	52	1.17 (0.60–2.27)	35	0.65 (0.33–1.29)	0.88 (0.39–2.02)
Q4	62	53	1.52 (0.79–2.92)	89	2.07 (1.14–3.77)	3.40 (1.47–7.83)
*P*_trend_			0.32		0.009	0.003
5-MTHF						
Q1 (low)	62	75	1.00 (ref)	61	1.00 (ref)	—
Q2	63	49	0.80 (0.43–1.47)	60	1.1 (0.61–1.96)	
Q3	61	42	0.74 (0.40–1.4)	41	0.77 (0.41–1.44)	
Q4	62	42	0.65 (0.35–1.21)	41	0.87 (0.47–1.60)	
*P*_trend_			0.16		0.44	
PLP						
Q1 (low)	63	89	1.00 (ref)	140	1.00 (ref)	1.00 (ref)
Q2	63	53	0.62 (0.34–1.10)	31	0.23 (0.13–0.42)	0.25 (0.13–0.50)
Q3	63	37	0.67 (0.36–1.26)	17	0.16 (0.08–0.32)	0.21 (0.10–0.46)
Q4	62	30	0.67 (0.34–1.33)	14	0.18 (0.09–0.38)	0.25 (0.11–0.55)
*P*_trend_			0.26		<0.0001	<0.0001

5-MTHF, 5-methyltetrahydrofolate; BE, Barrett's esophagus; BMI, body mass index; CI, confidence interval; EAC, esophageal adenocarcinoma; NSAID, nonsteroidal anti-inflammatory drug; OR, odds ratio; PLP, pyridoxal 5-phosphate (vitamin B6); SAH, S-adenosylhomocysteine; SAM, S-adenosylmethionine; tHcy, total homocysteine.

a Multimarker model includes all markers with a statistically significant linear trend for EAC risk in the single marker model except SAM/SAH ratio due to inclusion of SAM.

b Adjusted for sex (male/female), age (years), smoking status (current/previous/never), BMI 5 years prior, location (Northern Ireland/Republic of Ireland), education (years), occupation (manual/non-manual), alcohol intake (none, <median, ≥median), *Helicobacter pylori* infection (seropositive/seronegative), gastroesophageal reflux symptoms (ever/never), and regular NSAID use (ever/never).

In comparison with people in the lowest quartile, those with the highest plasma levels of circulating methionine, betaine, choline, and vitamin B6 (PLP) had a 62%–82% reduced risk of EAC (OR 0.35; 95% CI 0.19–0.65; OR 0.38; 95% CI 0.21–0.71; OR 0.30; 95% CI 0.16–0.57; OR 0.18; 95% CI 0.09–0.38 for methionine, betaine, choline, and vitamin B6 (PLP), respectively; Table 2). Increasing levels of cystathionine and SAM, however, were associated with a 2-fold to 3-fold increased risk of EAC (OR 2.07; 95% CI 1.14–3.77 and OR 3.04; 95% CI 1.56–5.93, respectively) for highest vs lowest levels. In a model that included all of the biomarkers associated with EAC risk, methionine, SAM, choline, cystathionine, and PLP were all independently and significantly associated with EAC risk whereas betaine and tHcy were no longer associated. In sensitivity analyses limited to individuals without regular GER symptoms, associations were similar to, or stronger than, those observed in the full study sample (see Supplementary Table 2, Supplementary Digital Content 1, http://links.lww.com/CTG/B325).

To evaluate the association between overall plasma levels of dietary methyl donors inversely associated with EAC risk (i.e., methionine, betaine and choline), the methyl replete score was evaluated (Table 3). Highest levels of circulating dietary methyl donors were associated with a nearly 70% reduced risk of EAC (OR 0.33; 95% CI 0.16–0.66), with a similar directionality for BE (OR 0.53; 95% CI 0.25–1.12), albeit not statistically significant. In stratified analyses by median PLP level, the observed association between methyl replete score and EAC risk was only evident among those with lower circulating PLP levels, although the EAC case sample size was small among those with high PLP levels. In the analysis stratified by *H. pylori* status, the inverse association between methyl replete score and EAC risk was most pronounced among those who were *H. pylori* seropositive vs seronegative.

**Table 3.** Associations between methyl replete score and risks of BE and EAC overall and stratified by PLP level, FINBAR

Methyl replete score	Case-control comparisons				
	Controls	BE cases		EAC cases	
	n	n	OR (95% CI)^a^	n	OR (95% CI)^a^
All participants^b^					
0 (lowest)	54	59	1.00 (ref)	87	1.00 (ref)
1	72	67	0.85 (0.47–1.54)	51	0.36 (0.20–0.64)
2	73	60	0.91 (0.50–1.68)	43	0.34 (0.19–0.61)
3	52	25	0.53 (0.25–1.12)	23	0.33 (0.16–0.66)
*P*_trend_			0.20		0.0001
PLP < median^b^					
0 (lowest)	54	41	1.00 (ref)	79	1.00 (ref)
1	72	45	0.96 (0.46–2.04)	40	0.36 (0.18–0.70)
2	73	38	1.10 (0.50–2.42)	33	0.32 (0.15–0.66)
3	52	18	0.68 (0.27–1.72)	18	0.35 (0.15–0.83)
*P*_trend_			0.62		0.002
PLP ≥ median^b^					
0 (lowest)	54	18	1.00 (ref)	6	1.00 (ref)
1	72	22	0.68 (0.24–1.93)	11	0.55 (0.13–2.23)
2	73	20	0.67 (0.24–1.88)	9	0.71 (0.19–2.64)
3	52	7	0.32 (0.08–1.25)	5	0.62 (0.14–2.74)
*P*_trend_			0.13		0.65
*H pylori* −					
0 (lowest)	22	30	1.00 (ref)	45	1.00 (ref)
1	27	27	0.87 (0.35–2.21)	19	0.34 (0.14–0.84)
2	35	33	0.93 (0.38–2.28)	23	0.31 (0.13–0.75)
3	16	10	0.66 (0.20–2.14)	12	0.65 (0.23–1.87)
*P*_trend_			0.65		0.09
*H pylori* +					
0 (lowest)	30	27	1.00 (ref)	40	1.00 (ref)
1	42	38	0.82 (0.37–1.83)	25	0.37 (0.17–0.79)
2	38	24	0.85 (0.36–2.04)	17	0.34 (0.15–0.77)
3	34	14	0.42 (0.16–1.16)	10	0.20 (0.07–0.53)
*P*_trend_			0.14		0.0005

BE, Barrett's esophagus; BMI, body mass index; CI, confidence interval; EAC, esophageal adenocarcinoma; NSAID, nonsteroidal anti-inflammatory drug; OR, odds ratio; PLP, pyridoxal 5-phosphate (vitamin B6).

a Adjusted for sex (male/female), age (years), smoking status (current/previous/never), BMI 5 years prior, location (Northern Ireland/Republic of Ireland), education (years), occupation (manual/nonmanual), alcohol intake (none, <median, ≥median), gastroesophageal reflux symptoms (ever/never), and regular NSAID use (ever/never).

b Additionally adjusted for *Helicobacter pylori* infection (seropositive/seronegative).

## DISCUSSION

In a large case-control study of long-segment BE and EAC, we found multiple relevant biomarkers of one-carbon metabolism to be significantly associated with the risk of EAC. Specifically, higher levels of plasma betaine, choline, vitamin B6, and methionine were associated with a significantly decreased likelihood of EAC, whereas higher levels of SAM and cystathionine were associated with a significantly higher likelihood of EAC. Furthermore, higher methyl donor status, as measured by the methyl replete score, was associated with an approximately 65% lower risk of EAC. The associations between these biomarkers and the methyl replete score and nondysplastic long-segment BE, however, were generally null. To our knowledge, this is the first study, to analyze the association between plasma one-carbon metabolism biomarkers and the risk of BE and the largest to analyze this association with EAC.

One-carbon metabolism is an umbrella term to describe the complex metabolic network that is responsible for the integration of single-carbon methyl units across a broad range of cellular processes. This network is comprised primarily of 2 interrelated cycles, the methionine cycle and the folate cycle. The methionine cycle and the various byproducts mediate a plethora of biological processes that are critical to maintaining homeostasis and preventing disease, including carcinogenesis. As the sole source of SAM, the methyl donor for nearly all methylation reactions in the body, methionine may mediate neoplastic risk and progression (36,37). Thus, aberrations in the methionine cycle might affect gene expression, be it through epigenetic modifications (DNA and histone methylation) or post-translational or post-transcriptional modifications.

Most studies evaluating the relationship of one-carbon metabolism with the risk of BE or EAC have solely analyzed dietary intakes of one-carbon metabolism cofactors and substrates. However, dietary intake may not fully reflect the intricate network of one-carbon metabolism pathways and the effect of genetic polymorphisms and epigenetic modifications on the available pool of one-carbon metabolism substrates and is also subject to potential recall biases. Plasma biomarkers integrate both dietary intake and individual metabolism and thus may more accurately represent the internal milieu compared with dietary intake alone (38). Only 1 study has previously analyzed plasma levels of one-carbon metabolism metabolites and EAC risk specifically (39). That study, which was a nested case:control study of the European Prospective Investigation into Cancer and Nutrition prospective cohort, reported no association between plasma levels of vitamins B2 (median among controls = 13.1 nmol/L), B6 (PLP; median among controls = 34.5 nmol/L), B9 (folate; median among controls = 12.9 nmol/L), methionine (median among controls = 25.1 μmol/L), or Hcy (median among controls = 10.2 μmol/L) and EAC risk or survival, based on 74 EAC cases. The authors did report a positive association between plasma Hcy level (median among controls = 10.2 μmol/L) and cancers of the oral cavity, gums, and oropharynx, but associations were null for esophageal squamous cell cancer (39). A population-based case-control study from China analyzed the association between plasma folate, vitamin B12, and tHcy levels and esophageal cancer risk but did not specify the histological type; this study reported that the highest quartile of vitamin B12 (>324.06 pmol/L) was associated with over 2-fold higher risk of esophageal cancer, but otherwise reported null associations for folate (median among controls = 12.76 nmol/L) and Hcy (median among controls = 9.50 μmol/L) (40). Because only postdiagnosis plasma samples were evaluated, the authors acknowledge the possibility of reverse causality underlying their findings but cited experimental and epidemiological studies supporting a positive association between elevated plasma vitamin B12 levels and other GI cancers (e.g., liver and gastric cancer) (40,41). In our study, higher plasma levels of the methyl sources, methionine, betaine, and choline were associated with decreased risk of EAC, as was PLP. Betaine and choline contribute to the one-carbon metabolism cycle as a source of methionine through a PLP-dependent pathway (42). We demonstrated a significant association between elevated cystathionine levels and BE/EAC risk compared with population controls which has not previously been investigated. Higher expression of cystathionine-beta-synthase, for which cystathionine is a hypothesized surrogate marker, is common in some cancers and related to survival (43). Interestingly, cystathionine-beta-synthase enzyme seems to be a highly recurrent target of epigenetic silencing in the CpG island methylator phenotype of gastric cancer (44); its role in progression to EAC is not clear.

We also observed that higher levels of SAM, the methyl donor, and the SAM/SAH ratio were associated with increased risk of EAC. Plasma concentrations of SAM are associated with global DNA methylation in tissues (21,45), and altered tissue DNA methylation patterns are a prominent feature of both BE and EAC. In almost all cases, DNA methylation occurs on a cytosine in a CpG dinucleotide, which is concentrated in promoter regions and therefore has the ability to profoundly affect gene expression. Several studies further suggest that these epigenetic modifications are critical early in the carcinogenesis pathway (8,46–49), which makes this a particularly promising biomarker for the detection of early-stage EAC and also therapeutic targets to potentially even halt the malignant transformation of BE. The exact interplay of hypermethylation vs hypomethylation in BE pathogenesis and progression to EAC remains to be elucidated. One suggested key early event has been GER leading to inflammation and accumulation of epigenetic changes, including DNA methylation-related silencing of tumor suppressor genes (46,50–52). More recent genome-wide analyses demonstrate significant global hypomethylation early in progression to EAC, even before frank invasion (46). Notably, SAM/SAH, also known as the methylation index, is often considered a marker of tissue methylation capacity (45). It is possible that higher tissue methylation capacity (SAM/SAH) is a marker of global hypomethylation, which is supported by increasing SAM/SAH being independently associated with EAC, and higher methyl donor status (i.e., higher methyl replete score) was also associated with lower EAC risk. The lack of an association with nondysplastic long-segment BE suggests plasma SAM, and SAM/SAH might have utility in identifying patients at highest risk of EAC. External validation studies are needed, as are studies correlating plasma SAM, SAH, and SAM/SAH with tissue level DNA-methylation patterns.

Apart from the novelty of this study, additional strengths include quality control for each of the plasma biomarkers tested, comprehensive dietary assessments, and covariate capture including GER symptoms, as well as rigorous analysis. Only individuals with EAC and long-segment BE that was histopathologically confirmed were included, which reduces misclassification (e.g., hiatal hernias may sometimes be misclassified as short segment BE). Restricting the BE cases to long-segment BE also enriches for a higher risk group at baseline as long-segment BE is associated with significantly higher risk of EAC progression vs short-segment BE (53), that we demonstrated null associations even for long-segment BE, but several positive associations for EAC enhances the clinical relevance of our findings for risk stratification. In addition to adjusting for clinical factors and relevant dietary intakes, we also adjusted for plasma levels of other one-carbon metabolism substrates in the multimarker model. Our study has some limitations. We cannot exclude reverse causation, and it is possible that biomarkers levels and the observed associations are due to the disease process. Owing to their diagnoses, individuals with BE or EAC may have made changes in their diet or lifestyle, which might affect circulating metabolite levels. We are also unable to comment on the generalizability of our findings outside of the Irish population. We were underpowered to assess associations among women.

In conclusion, we identified several biomarkers of one-carbon metabolism that are associated with EAC risk and have biological plausibility for promoting BE neoplastic progression. We are still in the early stages of understanding the intricacies of one-carbon metabolism in carcinogenesis and leveraging these pathways for diagnostic, therapeutic, and prognostic clinical use in other cancer types. As the first study to investigate plasma biomarkers of one-carbon metabolism and BE/EAC risk, this analysis extends our understanding and suggests that this pathway is relevant in esophageal carcinogenesis. Future studies validating our findings and investigating the predictive capability of these biomarkers when combined with individual clinical risk factors of high-risk BE and early EAC, are needed. When considering the massive burden of BE/EAC and the current trajectory, the public health and health economic implications of identifying precise noninvasive biomarkers and incorporating these into clinical practice are substantial.

## CONFLICTS OF INTEREST

**Guarantor of the article:** Martha J. Shrubsole, PhD.

**Specific author contributions:** L.A.A. and the FINBAR study group conceived and designed the FINBAR study. M.J.S., T.B., H.G.C., and C.Y. developed the study concept and designed the study in this manuscript. M.J.S., L.A.A., H.G.C., and T.B. acquired the data. M.A.H.D., X.Z., and M.J.S. conducted data analysis. M.J.S., S.C.S., M.A.H.D., and H.G.C. drafted the manuscript. All authors contributed to interpretation of data and critical revision of the manuscript for important intellectual content. MJS obtained funding.

**Financial support:** The study was supported by grant R03CA195660 from the National Cancer Institute to M.J.S. The FINBAR study was supported by funding from Cancer Focus Northern Ireland (formerly the Ulster Cancer Foundation), the Northern Ireland Research & Development office and the Health Research Board. H.G.C. is currently supported by a Cancer Research UK Career Establishment Award. S.C.S. is or was supported by Veteran's Affairs grant ICX002027A01 and an American Gastroenterological Association Research Scholar Award 2019. None of the funders had any role in the design, analysis or writing of this article.

**Potential competing interests:** Authors have no potential conflicts to disclose that are related to this manuscript. M.J.S. has received research funding support from Pfizer that is unrelated to this study.

**IRB approval:** This human subject research study was approved by the Research Ethics Committee of Queen's University Belfast, the Clinical Research Ethics Committee of Cork Teaching Hospitals, and the Research Ethics Committee Board of St. James's Hospital, Dublin. Written informed consent was obtained from all participants.

**Data availability:** The data that support the findings of this study are available from the corresponding author, M.J.S., upon reasonable request and when consistent with the informed consent of study participants and institutional policies of the participating sites.

**Study Highlights**

**WHAT IS KNOWN**

✓ The methionine cycle is the methyl donor for all methylation reactions.
✓ Epigenetic changes are common in Barrett's esophagus and esophageal adenocarcinoma (EAC).

**WHAT IS NEW HERE**

✓ Circulating levels of methionine, vitamin B6, and choline are associated with reduced EAC risk.
✓ Circulating levels of *S*-adenosylmethionine, homocysteine, and cystathionine are strongly associated with EAC risk.

## Supplementary Material

SUPPLEMENTARY MATERIAL

## ABBREVIATIONS:

BE: Barrett's esophagus
BMI: body mass index
CI: confidence interval
EAC: esophageal adenocarcinoma
FINBAR: Factors INfluencing the Barrett Adenocarcinoma Relationship study
GER: gastroesophageal reflux
OR: odds ratio
PLP: pyridoxal 5-phosphate (vitamin B6)
SAH: *S*-adenosylhomocysteine
SAM: *S*-adenosylmethionine
tHcy: total homocysteine
