# Supplementary material for: Plasma Metabolites of One-Carbon Metabolism Are Associated With Esophageal Adenocarcinoma in a Population-Based Study
Source: Clin Transl Gastroenterol. 2025 Jun 26;16(10):e00879. doi: 10.14309/ctg.0000000000000879 (PMC12543241; doi:10.14309/ctg.0000000000000879)
Supplement: SUPPLEMENTARY MATERIAL [file ct9-16-e00879-s001.docx]

**Supplemental Materials**

Plasma metabolites of one-carbon metabolism are associated with esophageal adenocarcinoma in a population-based study

**Supplemental Table 1· Sex differences and quartile ranges in biomarker levels among controls, FINBAR study.**

| **Plasma Biomarker** | **Mean ± SD** | | **p-value ^a^** |  | **Quartile (Q1) Range** | |
| --- | --- | --- | --- | --- | --- | --- |
|  | **Males** | **Females** |  |  | **Males** | **Females** |
| Methionine (µmol/L) | 26·4 ± 8·3 | 22·2 ± 8·9 | 0·004 |  | Q1: ≤ 20·8  Q2: 20·9-25·9  Q3: 26·0-30·3  Q4: ≥ 30·4 | Q1: ≤ 17·4  Q2: 17·5-21·2  Q3: 21·3-23·7  Q4: ≥ 23·8 |
| *S*-adenosylmethionine (SAM) (nmol/L) | 89·6 ± 36·5 | 87·9 ± 29·0 | 0·78 |  | Q1: ≤ 68·8  Q2: 69·8-86·5  Q3: 86·6-103·0  Q4: ≥ 103·1 | Q1: ≤ 68·0  Q2: 68·1-80·7  Q3: 80·8-96·0  Q4: ≥ 96·1 |
| *S*-adenosylhomocysteine (SAH) (nmol/L) | 31·9 ± 32·3 | 26·1 ± 11·8 | 0·27 |  | Q1: ≤ 21·5  Q2: 21·6-27·0  Q3: 27·1-34·5  Q4: ≥ 34·6 | Q1: ≤ 19·0  Q2: 19·1-23·3  Q3: 23·4-30·0  Q4: ≥ 30·1 |
| SAM/SAH ratio | 3·25 ± 1·21 | 3·69 ± 1·26 | 0·04 |  | Q1: ≤ 2·44  Q2: 2·44-3·28  Q3: 3·28-4·02  Q4: ≥ 4·03 | Q1: ≤ 3·00  Q2: 3·01-3·70  Q3: 3·71-4·16  Q4: ≥ 4·17 |
| Total homocysteine (tHcy) (µmol/L) | 12·7 ± 9·2 | 12·5 ± 4·5 | 0·92 |  | Q1: ≤ 8·9  Q2: 9·0-10·8  Q3: 10·9-13·8  Q4: ≥13·9 | Q1: ≤ 8·6  Q2: 8·7-11·9  Q3: 12·0-14·0  Q4: ≥14·1 |
| Betaine (µmol/L) | 49·7 ± 13·6 | 47·0 ± 10·7 | 0·24 |  | Q1: ≤ 40·5  Q2: 40·6-46·7  Q3: 46·8-57·1  Q4: ≥ 57·2 | Q1: ≤ 36·5  Q2: 36·6-47·8  Q3: 47·9-54·2  Q4: ≥ 54·3 |
| Choline (µmol/L) | 9·3 ± 3·4 | 9·2 ± 3·2 | 0·79 |  | Q1: ≤ 6·9  Q2: 7·0-8·8  Q3: 8·0-10·8  Q4: ≥ 10·9 | Q1: ≤ 7·6  Q2: 7·7-8·9  Q3: 9·0-10·0  Q4: ≥ 10·1 |
| Cystathionine (µmol/L) | 282 ± 241 | 26·4 ± 139 | 0·67 |  | Q1: ≤ 163·0  Q2: 164·0-220·0  Q3: 221·0-310·0  Q4: ≥ 311·0 | Q1: ≤ 151·0  Q2: 152·0-249·0  Q3: 250·0-322·0  Q4: ≥ 323·0 |
| 5-methyl tetrahydrofolate (MTHF) (nmol/L) | 15·5 ± 12·9 | 16·5 ± 13·2 | 0·67 |  | Q1: ≤ 7·6  Q2: 7·7-11·8  Q3: 11·9-18·3  Q4: ≥ 18·4 | Q1: ≤ 7·6  Q2: 7·7-13·4  Q3: 13·5-18·8  Q4: ≥ 18·9 |
| Pyridoxal 5′-phosphate (PLP) (nmol/L) | 82·6 ± 67·5 | 103 ± 132 | 0·15 |  | Q1: ≤ 45·6  Q2: 45·7-66·5  Q3: 67·5-100·0  Q4: ≥ 101·0 | Q1: ≤ 50·2  Q2: 50·3-69·7  Q3: 70·7-86·2  Q4: ≥ 86·3 |
| ^a^ Males compared to females calculated using the *t*-test. | | | |  |  |  |

**Supplemental Table 2 Associations between one-carbon metabolism biomarkers and risk of esophageal adenocarcinoma by male sex, age, and BMI strata, FINBAR.**

| **Biomarker and**  **Stratum** | **Quartile 1 (Low)** | |  | **Quartile 2** | |  | **Quartile 3** | |  | **Quartile 4 (High)** | | **P_trend_** |
| --- | --- | --- | --- | --- | --- | --- | --- | --- | --- | --- | --- | --- |
|  | **Case/**  **Control** | **OR (95%CI)** |  | **Case/**  **Control** | **OR (95%CI)** |  | **Case/**  **Control** | **OR (95%CI)** |  | **Case/**  **Control** | **OR (95%CI)** |  |
| **Methionine** |  |  |  |  |  |  |  |  |  |  |  |  |
| Males | 92/53 | 1·00 (ref) |  | 33/53 | 0·36 (0·19-0·68) |  | 22/53 | 0·27 (0·13-0·55) |  | 25/52 | 0·35 (0·17-0·68) | 0·0002 |
|  |  |  |  |  |  |  |  |  |  |  |  |  |
| <65 years | 48/23 | 1·00 (ref) |  | 13/34 | 0·19 (0·07-0·47) |  | 13/37 | 0·24 (0·09-0·60) |  | 23/33 | 0·44 (0·19-1·01) | 0·04 |
| ≥ 65 years | 61/40 | 1·00 (ref) |  | 24/29 | 0·56 (0·25-1·24) |  | 12/26 | 0·33 (0·13-0·87) |  | 10/29 | 0·19 (0·06-0·57) | 0·0008 |
|  |  |  |  |  |  |  |  |  |  |  |  |  |
| BMI <25 | 22/22 | 1·00 (ref) |  | 7/14 | 0·84 (0·24-2·98) |  | 6/16 | 0·49 (0·12-2·04) |  | 11/26 | 0·58 (0·18-1·85) | 0·25 |
| BMI 25 to <30 | 53/33 | 1·00 (ref) |  | 18/27 | 0·37 (0·15-0·91) |  | 12/34 | 0·21 (0·08-0·56) |  | 12/28 | 0·24 (0·09-0·65) | 0·0005 |
| BMI ≥ 30 | 33/8 | 1·00 (ref) |  | 12/21 | 0·10 (0·03-0·38) |  | 7/12 | 0·13 (0·03-0·61) |  | 10/5 | 0·23 (0·05-1·06) | 0·04 |
|  |  |  |  |  |  |  |  |  |  |  |  |  |
| *H· pylori* - | 49/19 | 1·00 (ref) |  | 19/30 | 0·32 (0·13-0·77) |  | 11/28 | 0·23 (0·08-0·64) |  | 20/23 | 0·43 (0·16-1·13) | 0·04 |
| *H· pylori* + | 51/43 | 1·00 (ref) |  | 16/30 | 0·32 (0·14-0·72) |  | 13/34 | 0·28 (0·12-0·67) |  | 12/37 | 0·28 (0·12-0·67) | 0·0005 |
|  |  |  |  |  |  |  |  |  |  |  |  |  |
| Reflux - | 5/48 | 1·00 (ref) |  | 19/50 | 0·31 (0·15-0·65) |  | 10/51 | 0·18 (0·08-0·44) |  | 18/52 | 0·32 (0·15-0·68) | 0·0003 |
| Reflux + | 51/15 | 1·00 (ref) |  | 18/13 | 0·39 (0·14-1·10) |  | 15/11 | 0·42 (0·14-1·25) |  | 15/10 | 0·48 (0·15-1·52) | 0·11 |
|  |  |  |  |  |  |  |  |  |  |  |  |  |
| Never smoker | 25/21 | 1·00 (ref) |  | 6/32 | 0·09(0·03-0·31) |  | 4/18 | 0·15(0·04-0·62) |  | 6/25 | 0·2(0·06-0·73) | 0·0037 |
| Former smoker | 47/29 | 1·00 (ref) |  | 19/21 | 0·71(0·27-1·82) |  | 11/31 | 0·25(0·09-0·69) |  | 14/24 | 0·46(0·17-1·26) | 0·0238 |
| Current smoker | 35/11 | 1·00 (ref) |  | 11/9 | 0·47(0·14-1·56) |  | 10/11 | 0·48(0·14-1·66) |  | 12/13 | 0·4(0·12-1·29) | 0·0994 |
|  |  |  |  |  |  |  |  |  |  |  |  |  |
| No alcohol | 36/18 | 1·00 (ref) |  | 9/16 | 0·28(0·08-1·01) |  | 4/17 | 0·12(0·03-0·5) |  | 10/16 | 0·34(0·09-1·2) | 0·0110 |
| Alcohol < median (20 g/day) | 44/26 | 1·00 (ref) |  | 19/26 | 0·51(0·22-1·2) |  | 15/18 | 0·68(0·26-1·81) |  | 13/18 | 0·48(0·18-1·28) | 0·1507 |
| Alcohol ≥ median | 29/19 | 1·00 (ref) |  | 9/21 | 0·24(0·07-0·85) |  | 6/28 | 0·19(0·05-0·76) |  | 10/28 | 0·31(0·1-1) | 0·0210 |
|  |  |  |  |  |  |  |  |  |  |  |  |  |
| **SAM** |  |  |  |  |  |  |  |  |  |  |  |  |
| Males | 23/53 | 1·00 (ref) |  | 41/53 | 2·03 (0·99-4·16) |  | 40/53 | 1·40 (0·67-2·94) |  | 68/53 | 2·42 (1·17-5·03) | 0·06 |
|  |  |  |  |  |  |  |  |  |  |  |  |  |
| <65 years | 19/39 | 1·00 (ref) |  | 26/36 | 1·77 (0·75-4·20) |  | 18/33 | 1·15 (0·46-2·87) |  | 34/19 | 3·04 (1·19-7·80) | 0·06 |
| ≥ 65 years | 6/24 | 1·00 (ref) |  | 22/27 | 3·02 (0·93-9·79) |  | 26/30 | 2·64 (0·80-8·67) |  | 53/43 | 4·52 (1·47-13·93) | 0·02 |
|  |  |  |  |  |  |  |  |  |  |  |  |  |
| BMI <25 | 6/26 | 1·00 (ref) |  | 11/20 | 3·42 (0·84-14·02) |  | 6/13 | 1·95 (0·38-9·94) |  | 23/13 | 8·72 (2·03-37·54) | 0·006 |
| BMI 25 to <30 | 11/29 | 1·00 (ref) |  | 28/34 | 2·74 (0·97-7·76) |  | 22/32 | 1·51 (0·50-4·58) |  | 34/27 | 2·29 (0·77-6·83) | 0·38 |
| BMI ≥ 30 | 8/7 | 1·00 (ref) |  | 9/9 | 0·64 (0·14-3·00) |  | 16/17 | 0·63 (0·15-2·54) |  | 29/16 | 1·17 (0·30-4·64) | 0·69 |
|  |  |  |  |  |  |  |  |  |  |  |  |  |
| *H· pylori* - | 11/24 | 1·00 (ref) |  | 28/26 | 3·12 (1·13-8·61) |  | 21/25 | 1·44 (0·52-4·04) |  | 39/25 | 1·90 (0·69-5·28) | 0·60 |
| *H· pylori* + | 12/39 | 1·00 (ref) |  | 16/36 | 1·44 (0·56-3·70) |  | 21/36 | 1·58 (0·60-4·14) |  | 43/33 | 4·21 (1·7-10·4) | 0·001 |
|  |  |  |  |  |  |  |  |  |  |  |  |  |
| Reflux - | 17/51 | 1·00 (ref) |  | 25/56 | 1·45 (0·64-3·31) |  | 25/47 | 1·84 (0·78-4·36) |  | 38/47 | 2·8 0(1·24-6·29) | 0·01 |
| Reflux + | 8/11 | 1·00 (ref) |  | 23/7 | 5·14 (1·36-19·39) |  | 19/16 | 1·68 (0·48-5·80) |  | 44/15 | 5·29 (1·49-18·81) | 0·06 |
|  |  |  |  |  |  |  |  |  |  |  |  |  |
| Never smoker | 6/22 | 1·00 (ref) |  | 10/25 | 1·8(0·48-6·72) |  | 8/34 | 1·02(0·26-3·97) |  | 17/15 | 6·65(1·61-27·45 | 0·0201 |
| Former smoker | 8/24 | 1·00 (ref) |  | 20/25 | 3·26(1·03-10·32 |  | 24/24 | 1·78(0·57-5·57) |  | 39/32 | 3·25(1·07-9·9) | 0·1343 |
| Current smoker | 11/15 | 1·00 (ref) |  | 17/12 | 1·81(0·54-5·99) |  | 11/4 | 2·8(0·57-13·82) |  | 29/13 | 3·26(0·93-11·42 | 0·0684 |
|  |  |  |  |  |  |  |  |  |  |  |  |  |
| No alcohol | 2/9 | 1·00 (ref) |  | 12/17 | 2·91(0·45-18·64 |  | 15/24 | 1·84(0·28-12·08 |  | 30/17 | 7·01(1·14-42·95 | 0·0396 |
| Alcohol < median (20 g/day) | 14/29 | 1·00 (ref) |  | 18/20 | 1·85(0·68-5·05) |  | 22/18 | 3·12(1·11-8·83) |  | 37/21 | 3·97(1·45-10·9) | 0·0030 |
| Alcohol ≥ median | 9/25 | 1·00 (ref) |  | 18/26 | 2·71(0·81-9·07) |  | 7/21 | 0·57(0·13-2·42) |  | 20/24 | 1·26(0·35-4·46) | 0·6652 |
|  |  |  |  |  |  |  |  |  |  |  |  |  |
| **SAH** |  |  |  |  |  |  |  |  |  |  |  |  |
| Males | 54/53 | 1·00 (ref) |  | 43/52 | 0·61 (0·31-1·18) |  | 32/53 | 0·53 (0·26-1·06) |  | 52/43 | 0·77 (0·39-1·55) | 0·42 |
|  |  |  |  |  |  |  |  |  |  |  |  |  |
| <65 years | 40/33 | 1·00 (ref) |  | 21/37 | 0·38 (0·16-0·87) |  | 13/29 | 0·39 (0·15-1·00) |  | 23/27 | 0·62 (0·25-1·51) | 0·27 |
| ≥ 65 years | 19/30 | 1·00 (ref) |  | 27/25 | 1·11 (0·42-2·99) |  | 28/34 | 0·85 (0·32-2·26) |  | 33/35 | 1·35 (0·51-3·56) | 0·63 |
|  |  |  |  |  |  |  |  |  |  |  |  |  |
| BMI <25 | 18/25 | 1·00 (ref) |  | 9/15 | 0·70 (0·20-2·47) |  | 7/21 | 0·48 (0·13-1·72) |  | 12/17 | 1·17 (0·34-4·09) | 0·94 |
| BMI 25 to <30 | 27/30 | 1·00 (ref) |  | 25/34 | 0·42 (0·16-1·09) |  | 20/26 | 0·77 (0·29-2·03) |  | 23/31 | 0·56 (0·21-1·48) | 0·47 |
| BMI ≥ 30 | 14/7 | 1·00 (ref) |  | 14/13 | 0·49 (0·12-2·07) |  | 14/16 | 0·26 (0·06-1·10) |  | 20/13 | 0·67 (0·16-2·75) | 0·58 |
|  |  |  |  |  |  |  |  |  |  |  |  |  |
| *H· pylori* - | 31/24 | 1·00 (ref) |  | 29/24 | 0·55 (0·22-1·36) |  | 18/26 | 0·38 (0·14-1·03) |  | 21/26 | 0·39 (0·15-1·03) | 0·04 |
| *H· pylori* + | 25/38 | 1·00 (ref) |  | 17/38 | 0·57 (0·23-1·37) |  | 19/35 | 0·85 (0·36-2·02) |  | 31/32 | 1·40 (0·59-3·33) | 0·27 |
|  |  |  |  |  |  |  |  |  |  |  |  |  |
| Reflux - | 33/56 | 1·00 (ref) |  | 31/45 | 1·02 (0·49-2·14) |  | 14/51 | 0·50 (0·22-1·15) |  | 27/48 | 0·98 (0·46-2·08) | 0·56 |
| Reflux + | 26/7 | 1·00 (ref) |  | 17/17 | 0·15 (0·05-0·53) |  | 27/12 | 0·46 (0·12-1·67) |  | 29/13 | 0·46 (0·12-1·75) | 0·74 |
|  |  |  |  |  |  |  |  |  |  |  |  |  |
| Never smoker | 11/30 | 1·00 (ref) |  | 7/20 | 0·81(0·24-2·69) |  | 9/28 | 0·79(0·24-2·67) |  | 14/18 | 2·18(0·64-7·41) | 0·2704 |
| Former smoker | 20/22 | 1·00 (ref) |  | 22/27 | 0·6(0·22-1·65) |  | 24/25 | 0·66(0·24-1·88) |  | 25/30 | 0·64(0·23-1·8) | 0·5172 |
| Current smoker | 28/9 | 1·00 (ref) |  | 16/14 | 0·38(0·11-1·23) |  | 8/9 | 0·26(0·07-1·03) |  | 16/12 | 0·31(0·09-1·1) | 0·0580 |
|  |  |  |  |  |  |  |  |  |  |  |  |  |
| No alcohol | 11/17 | 1·00 (ref) |  | 15/13 | 0·87(0·23-3·3) |  | 14/17 | 0·85(0·23-3·14) |  | 19/20 | 0·96(0·28-3·35) | 0·9598 |
| Alcohol < median (20 g/day) | 24/23 | 1·00 (ref) |  | 25/25 | 0·91(0·37-2·28) |  | 19/22 | 0·93(0·36-2·45) |  | 23/18 | 1·2(0·43-3·34) | 0·7589 |
| Alcohol ≥ median | 24/23 | 1·00 (ref) |  | 8/24 | 0·18(0·05-0·65) |  | 8/24 | 0·16(0·04-0·61) |  | 14/24 | 0·32(0·1-1·06) | 0·0508 |
|  |  |  |  |  |  |  |  |  |  |  |  |  |
| **SAM/SAH ratio** |  |  |  |  |  |  |  |  |  |  |  |  |
| Males | 18/53 | 1·00 (ref) |  | 31/53 | 1·30 (0·59-2·84) |  | 53/53 | 2·47 (1·16-5·29) |  | 70/52 | 3·09 (1·47-6·52) | 0·0007 |
|  |  |  |  |  |  |  |  |  |  |  |  |  |
| <65 years | 12/37 | 1·00 (ref) |  | 17/31 | 1·20 (0·45-3·19) |  | 23/27 | 1·89 (0·72-4·95) |  | 45/31 | 3·29 (1·34-8·10) | 0·004 |
| ≥ 65 years | 16/26 | 1·00 (ref) |  | 26/31 | 1·90 (0·69-5·26) |  | 33/31 | 2·22 (0·80-6·15) |  | 32/31 | 2·32 (0·82-6·55) | 0·14 |
|  |  |  |  |  |  |  |  |  |  |  |  |  |
| BMI <25 | 9/22 | 1·00 (ref) |  | 5/16 | 0·69 (0·15-3·27) |  | 8/20 | 2·35 (0·54-10·24) |  | 24/20 | 2·78 (0·76-10·18) | 0·04 |
| BMI 25 to <30 | 9/26 | 1·00 (ref) |  | 23/33 | 1·34 (0·44-4·08) |  | 30/32 | 1·56 (0·50-4·89) |  | 33/30 | 2·20 (0·72-6·76) | 0·15 |
| BMI ≥ 30 | 9/14 | 1·00 (ref) |  | 15/13 | 1·43 (0·38-5·40) |  | 18/11 | 1·72 (0·47-6·34) |  | 20/11 | 3·62 (0·92-14·33) | 0·07 |
|  |  |  |  |  |  |  |  |  |  |  |  |  |
| *H· pylori* - | 14/26 | 1·00 (ref) |  | 17/29 | 0·90 (0·33-2·48) |  | 29/23 | 2·72 (0·98-7·54) |  | 39/22 | 3·21 (1·19-8·67) | 0·005 |
| *H· pylori* + | 12/34 | 1·00 (ref) |  | 21/32 | 2·01 (0·76-5·26) |  | 23/39 | 1·69 (0·65-4·38) |  | 36/38 | 2·53 (1·00-6·41) | 0·09 |
|  |  |  |  |  |  |  |  |  |  |  |  |  |
| Reflux - | 12/52 | 1·00 (ref) |  | 27/46 | 2·10 (0·88-5·03) |  | 26/51 | 2·22 (0·91-5·41) |  | 40/51 | 3·25 (1·41-7·51) | 0·009 |
| Reflux + | 16/10 | 1·00 (ref) |  | 16/16 | 0·79 (0·24-2·60) |  | 30/12 | 2·03 (0·62-6·65) |  | 37/11 | 2·71 (0·78-9·38) | 0·03 |
|  |  |  |  |  |  |  |  |  |  |  |  |  |
| Never smoker | 9/21 | 1·00 (ref) |  | 6/24 | 0·94(0·23-3·8) |  | 10/29 | 1·08(0·29-3·96) |  | 16/22 | 3·06(0·84-11·13 | 0·0614 |
| Former smoker | 11/27 | 1·00 (ref) |  | 22/26 | 2·88(0·95-8·74) |  | 30/23 | 5·13(1·64-16·07 |  | 28/28 | 3·63(1·18-11·22 | 0·0288 |
| Current smoker | 8/14 |  |  | 15/11 | 1·42(0·37-5·54) |  | 14/7 | 3·06(0·69-13·47 |  | 31/12 | 4·86(1·36-17·4) | 0·0070 |
|  |  |  |  |  |  |  |  |  |  |  |  |  |
| No alcohol | 11/12 | 1·00 (ref) |  | 14/22 | 0·75(0·2-2·79) |  | 15/15 | 1·41(0·33-5·93) |  | 19/18 | 1·39(0·35-5·49) | 0·3884 |
| Alcohol < median (20 g/day) | 11/25 | 1·00 (ref) |  | 20/20 | 1·89(0·65-5·49) |  | 27/17 | 3·66(1·27-10·55 |  | 33/26 | 2·38(0·87-6·55) | 0·0704 |
| Alcohol ≥ median | 6/26 | 1·00 (ref) |  | 9/20 | 1·9(0·42-8·73) |  | 14/31 | 1·61(0·37-6·97) |  | 25/18 | 9·55(2·34-38·88) | 0·0009 |
|  |  |  |  |  |  |  |  |  |  |  |  |  |
| **thcy** |  |  |  |  |  |  |  |  |  |  |  |  |
| Males | 30/53 | 1·00 (ref) |  | 26/53 | 0·68 (0·32-1·48) |  | 51/53 | 1·18 (0·60-2·33) |  | 65/52 | 1·50 (0·76-2·97) | 0·08 |
|  |  |  |  |  |  |  |  |  |  |  |  |  |
| <65 years | 18/37 | 1·00 (ref) |  | 19/37 | 1·04 (0·41-2·60) |  | 35/34 | 1·87 (0·81-4·35) |  | 25/19 | 2·30 (0·90-5·86) | 0·03 |
| ≥ 65 years | 15/26 | 1·00 (ref) |  | 13/26 | 0·58 (0·19-1·76) |  | 25/29 | 0·93 (0·34-2·52) |  | 54/43 | 1·59 (0·63-4·02) | 0·11 |
|  |  |  |  |  |  |  |  |  |  |  |  |  |
| BMI <25 | 9/15 | 1·00 (ref) |  | 13/28 | 0·70 (0·19-2·61) |  | 9/19 | 0·64 (0·16-2·50) |  | 15/16 | 1·47 (0·37-5·93) | 0·58 |
| BMI 25 to <30 | 15/33 | 1·00 (ref) |  | 11/24 | 0·94 (0·31-2·85) |  | 33/30 | 1·77 (0·68-4·62) |  | 36/35 | 1·55 (0·58-4·13) | 0·21 |
| BMI ≥ 30 | 8/13 | 1·00 (ref) |  | 8/11 | 1·33 (0·30-6·00) |  | 18/14 | 2·09 (0·52-8·46) |  | 28/11 | 4·76 (1·22-18·66) | 0·01 |
|  |  |  |  |  |  |  |  |  |  |  |  |  |
| *H· pylori* - | 19/28 | 1·00 (ref) |  | 13/25 | 0·54 (0·19-1·53) |  | 31/26 | 1·68 (0·68-4·17) |  | 36/21 | 1·55 (0·61-3·93) | 0·12 |
| *H· pylori* + | 12/33 | 1·00 (ref) |  | 17/37 | 1·35 (0·50-3·61) |  | 25/37 | 1·54 (0·60-3·97) |  | 38/37 | 2·39 (0·95-6·01) | 0·04 |
|  |  |  |  |  |  |  |  |  |  |  |  |  |
| Reflux - | 22/52 | 1·00 (ref) |  | 13/51 | 0·66 (0·28-1·56) |  | 32/53 | 0·92 (0·43-1·96) |  | 38/45 | 1·75 (0·83-3·67) | 0·10 |
| Reflux + | 11/10 | 1·00 (ref) |  | 19/12 | 1·71 (0·46-6·42) |  | 28/10 | 3·54 (0·96-12·96) |  | 41/17 | 2·97 (0·83-10·58 | 0·07 |
|  |  |  |  |  |  |  |  |  |  |  |  |  |
| Never smoker | 5/24 | 1·00 (ref) |  | 8/23 | 1·53(0·37-6·27) |  | 12/24 | 2·43(0·65-9·08) |  | 16/25 | 3·72(0·92-15·05 | 0·0390 |
| Former smoker | 14/24 | 1·00 (ref) |  | 13/29 | 0·95(0·31-2·9) |  | 25/26 | 1·78(0·63-5·05) |  | 39/26 | 2·76(1·01-7·56) | 0·0188 |
| Current smoker | 13/10 | 1·00 (ref) |  | 10/11 | 0·51(0·13-2·05) |  | 22/13 | 1·13(0·33-3·85) |  | 23/10 | 1·79(0·48-6·67) | 0·2533 |
|  |  |  |  |  |  |  |  |  |  |  |  |  |
| No alcohol | 9/16 | 1·00 (ref) |  | 8/20 | 0·31(0·07-1·34) |  | 15/14 | 1·08(0·27-4·3) |  | 27/17 | 2·17(0·6-7·84) | 0·0389 |
| Alcohol < median (20 g/day) | 15/16 | 1·00 (ref) |  | 14/21 | 0·82(0·27-2·44) |  | 29/30 | 0·97(0·36-2·64) |  | 33/21 | 1·89(0·68-5·28) | 0·1544 |
| Alcohol ≥ median | 9/31 | 1·00 (ref) |  | 10/22 | 2·63(0·68-10·11 |  | 16/19 | 2·96(0·86-10·16 |  | 19/24 | 1·36(0·39-4·74) | 0·5714 |
|  |  |  |  |  |  |  |  |  |  |  |  |  |
| **Betaine** |  |  |  |  |  |  |  |  |  |  |  |  |
| Males | 84/53 | 1·00 (ref) |  | 31/53 | 0·35 (0·18-0·66) |  | 34/53 | 0·50 (0·26-0·96) |  | 23/52 | 0·30 (0·15-0·62) | 0·001 |
|  |  |  |  |  |  |  |  |  |  |  |  |  |
| <65 years | 42/35 | 1·00 (ref) |  | 17/32 | 0·45 (0·19-1·04) |  | 19/29 | 0·60 (0·25-1·40) |  | 19/31 | 0·44 (0·18-1·07) | 0·09 |
| ≥ 65 years | 52/28 | 1·00 (ref) |  | 21/31 | 0·28 (0·12-0·67) |  | 17/34 | 0·29 (0·11-0·73) |  | 17/31 | 0·27 (0·11-0·68) | 0·003 |
|  |  |  |  |  |  |  |  |  |  |  |  |  |
| BMI <25 | 15/12 | 1·00 (ref) |  | 10/20 | 0·30 (0·08-1·14) |  | 10/24 | 0·38 (0·10-1·40) |  | 11/22 | 0·36 (0·09-1·41) | 0·21 |
| BMI 25 to <30 | 41/32 | 1·00 (ref) |  | 20/27 | 0·63 (0·26-1·52) |  | 19/36 | 0·60 (0·25-1·42) |  | 15/27 | 0·46 (0·17-1·25) | 0·10 |
| BMI ≥ 30 | 38/19 | 1·00 (ref) |  | 7/15 | 0·13 (0·03-0·46) |  | 7/3 | 0·60 (0·09-4·12) |  | 10/12 | 0·19 (0·05-0·72) | 0·02 |
|  |  |  |  |  |  |  |  |  |  |  |  |  |
| *H· pylori* - | 49/23 | 1·00 (ref) |  | 16/29 | 0·26 (0·10-0·63) |  | 16/29 | 0·43 (0·17-1·07) |  | 18/19 | 0·69 (0·26-1·83) | 0·30 |
| *H· pylori* + | 43/36 | 1·00 (ref) |  | 20/33 | 0·54 (0·25-1·21) |  | 16/32 | 0·48 (0·21-1·11) |  | 13/43 | 0·24 (0·10-0·56) | 0·001 |
|  |  |  |  |  |  |  |  |  |  |  |  |  |
| Reflux - | 47/44 | 1·00 (ref) |  | 22/52 | 0·37 (0·18-0·78) |  | 19/51 | 0·38 (0·18-0·81) |  | 17/54 | 0·30 (0·14-0·67) | 0·002 |
| Reflux + | 47/19 | 1·00 (ref) |  | 16/11 | 0·42 (0·15-1·17) |  | 17/12 | 0·55 (0·19-1·58) |  | 19/7 | 0·70 (0·23-2·15) | 0·34 |
|  |  |  |  |  |  |  |  |  |  |  |  |  |
| Never smoker | 20/29 | 1·00 (ref) |  | 9/21 | 0·51(0·16-1·61) |  | 4/24 | 0·26(0·07-0·98) |  | 8/22 | 0·51(0·15-1·7) | 0·1124 |
| Former smoker | 46/24 | 1·00 (ref) |  | 14/29 | 0·21(0·08-0·55) |  | 18/27 | 0·32(0·12-0·83) |  | 13/25 | 0·32(0·11-0·87) | 0·0127 |
| Current smoker | 26/10 | 1·00 (ref) |  | 14/11 | 0·38(0·12-1·23) |  | 13/10 | 0·62(0·17-2·22) |  | 15/13 | 0·3(0·09-1·04) | 0·0992 |
|  |  |  |  |  |  |  |  |  |  |  |  |  |
| No alcohol | 30/16 | 1·00 (ref) |  | 11/16 | 0·34(0·1-1·16) |  | 9/21 | 0·24(0·07-0·85) |  | 9/14 | 0·32(0·09-1·14) | 0·0294 |
| Alcohol < median (20 g/day) | 44/20 | 1·00 (ref) |  | 16/30 | 0·18(0·07-0·45) |  | 13/18 | 0·34(0·13-0·92) |  | 18/20 | 0·35(0·13-0·93) | 0·0394 |
| Alcohol ≥ median | 20/27 | 1·00 (ref) |  | 11/17 | 1·09(0·34-3·5) |  | 14/24 | 1·01(0·33-3·06) |  | 9/28 | 0·61(0·17-2·2) | 0·5023 |
|  |  |  |  |  |  |  |  |  |  |  |  |  |
| **Choline** |  |  |  |  |  |  |  |  |  |  |  |  |
| Males | 72/53 | 1·00 (ref) |  | 35/53 | 0·45 (0·23-0·86) |  | 36/54 | 0·43 (0·22-0·81) |  | 29/51 | 0·32 (0·16-0·65) | 0·0009 |
|  |  |  |  |  |  |  |  |  |  |  |  |  |
| <65 years | 44/37 | 1·00 (ref) |  | 19/36 | 0·44 (0·19-1·01) |  | 20/31 | 0·66 (0·29-1·52) |  | 14/23 | 0·35 (0·14-0·89) | 0·04 |
| ≥ 65 years | 45/26 | 1·00 (ref) |  | 20/28 | 0·29 (0·11-0·74) |  | 20/32 | 0·22 (0·08-0·56) |  | 22/38 | 0·22 (0·09-0·56) | 0·001 |
|  |  |  |  |  |  |  |  |  |  |  |  |  |
| BMI <25 | 24/22 | 1·00 (ref) |  | 9/16 | 0·74 (0·22-2·46) |  | 6/19 | 0·58 (0·15-2·27) |  | 7/19 | 0·45 (0·12-1·65) | 0·22 |
| BMI 25 to <30 | 35/29 | 1·00 (ref) |  | 19/33 | 0·41 (0·16-1·04) |  | 22/29 | 0·66 (0·27-1·60) |  | 19/31 | 0·34 (0·12-0·91) | 0·07 |
| BMI ≥ 30 | 30/11 | 1·00 (ref) |  | 11/13 | 0·33 (0·09-1·21) |  | 12/14 | 0·22 (0·06-0·81) |  | 9/11 | 0·21 (0·06-0·79) | 0·008 |
|  |  |  |  |  |  |  |  |  |  |  |  |  |
| *H· pylori* - | 46/27 | 1·00 (ref) |  | 17/27 | 0·40 (0·16-0·99) |  | 19/29 | 0·40 (0·17-0·96) |  | 17/17 | 0·41 (0·15-1·07) | 0·03 |
| *H· pylori* + | 39/34 | 1·00 (ref) |  | 18/36 | 0·42 (0·18-0·97) |  | 19/31 | 0·50 (0·21-1·19) |  | 16/43 | 0·23 (0·10-0·55) | 0·002 |
|  |  |  |  |  |  |  |  |  |  |  |  |  |
| Reflux - | 46/50 | 1·00 (ref) |  | 26/51 | 0·58 (0·28-1·20) |  | 21/50 | 0·52 (0·25-1·10) |  | 12/50 | 0·24 (0·10-0·56) | 0·001 |
| Reflux + | 43/12 | 1·00 (ref) |  | 13/13 | 0·18 (0·06-0·59) |  | 19/13 | 0·28 (0·09-0·87) |  | 24/11 | 0·35 (0·11-1·09) | 0·08 |
|  |  |  |  |  |  |  |  |  |  |  |  |  |
| Never smoker | 19/22 | 1·00 (ref) |  | 8/28 | 0·42(0·14-1·28) |  | 7/26 | 0·47(0·15-1·54) |  | 7/20 | 0·43(0·12-1·5) | 0·1457 |
| Former smoker | 36/23 | 1·00 (ref) |  | 17/26 | 0·23(0·08-0·64) |  | 19/26 | 0·27(0·1-0·73) |  | 19/30 | 0·23(0·08-0·62) | 0·0045 |
| Current smoker | 32/14 | 1·00 (ref) |  | 13/9 | 0·56(0·17-1·83) |  | 14/11 | 0·54(0·17-1·73) |  | 9/10 | 0·27(0·07-1·02) | 0·0565 |
|  |  |  |  |  |  |  |  |  |  |  |  |  |
| No alcohol | 31/9 | 1·00 (ref) |  | 8/19 | 0·21(0·05-0·81) |  | 7/19 | 0·14(0·03-0·57) |  | 13/20 | 0·15(0·04-0·53) | 0·0020 |
| Alcohol < median (20 g/day) | 36/22 | 1·00 (ref) |  | 19/29 | 0·46(0·19-1·1) |  | 18/20 | 0·64(0·25-1·63) |  | 18/17 | 0·49(0·18-1·3) | 0·1714 |
| Alcohol ≥ median | 22/32 | 1·00 (ref) |  | 12/16 | 0·81(0·25-2·61) |  | 15/24 | 0·62(0·2-1·9) |  | 5/24 | 0·15(0·03-0·67) | 0·0252 |
|  |  |  |  |  |  |  |  |  |  |  |  |  |
| **Cystathionine** |  |  |  |  |  |  |  |  |  |  |  |  |
| Males | 39/54 | 1·00 (ref) |  | 28/52 | 0·64 (0·31-1·32) |  | 30/53 | 0·57 (0·27-1·20) |  | 75/52 | 1·93 (1·00-3·73) | 0·02 |
|  |  |  |  |  |  |  |  |  |  |  |  |  |
| <65 years | 26/43 | 1·00 (ref) |  | 22/32 | 1·32 (0·55-3·18) |  | 9/26 | 0·52 (0·18-1·49) |  | 40/26 | 2·56 (1·11-5·93) | 0·05 |
| ≥ 65 years | 18/21 | 1·00 (ref) |  | 14/30 | 0·41 (0·14-1·25) |  | 26/37 | 0·75 (0·28-20·3) |  | 49/36 | 2·02 (0·79-5·17) | 0·02 |
|  |  |  |  |  |  |  |  |  |  |  |  |  |
| BMI <25 | 11/27 | 1·00 (ref) |  | 8/15 | 0·76 (0·18-3·16) |  | 5/20 | 0·54 (0·12-2·50) |  | 22/16 | 5·63 (1·58-20·06) | 0·01 |
| BMI 25 to <30 | 23/29 | 1·00 (ref) |  | 20/35 | 0·87 (0·33-2·29) |  | 14/27 | 0·59 (0·21-1·69) |  | 38/31 | 1·97 (0·78-4·96) | 0·16 |
| BMI ≥ 30 | 10/7 | 1·00 (ref) |  | 8/11 | 0·82 (0·17-3·84) |  | 16/16 | 0·68 (0·15-3·06) |  | 28/15 | 1·09 (0·26-4·60) | 0·80 |
|  |  |  |  |  |  |  |  |  |  |  |  |  |
| *H· pylori* - | 21/21 | 1·00 (ref) |  | 19/29 | 0·52 (0·19-1·38) |  | 17/28 | 0·43 (0·16-1·18) |  | 42/22 | 1·59 (0·63-4·01) | 0·22 |
| *H· pylori* + | 18/43 | 1·00 (ref) |  | 14/31 | 1·03 (0·41-2·63) |  | 15/34 | 0·94 (0·36-2·47) |  | 45/36 | 2·88 (1·25-6·67) | 0·008 |
|  |  |  |  |  |  |  |  |  |  |  |  |  |
| Reflux - | 21/51 | 1·00 (ref) |  | 20/52 | 0·90 (0·38-2·09) |  | 16/50 | 0·75 (0·30-1·85) |  | 48/48 | 2·56 (1·2-5·47) | 0·007 |
| Reflux + | 23/12 | 1·00 (ref) |  | 16/10 | 0·62 (0·19-2·00) |  | 19/13 | 0·53 (0·17-1·62) |  | 41/14 | 1·69 (0·56-5·16) | 0·34 |
|  |  |  |  |  |  |  |  |  |  |  |  |  |
| Never smoker | 8/25 | 1·00 (ref) |  | 7/21 | 1·15(0·3-4·4) |  | 6/29 | 0·69(0·16-2·91) |  | 20/21 | 4·55(1·28-16·1) | 0·0136 |
| Former smoker | 17/22 | 1·00 (ref) |  | 16/31 | 0·72(0·25-2·05) |  | 20/26 | 0·66(0·23-1·92) |  | 38/26 | 2·26(0·84-6·12) | 0·0606 |
| Current smoker | 19/14 | 1·00 (ref) |  | 12/9 | 0·64(0·17-2·37) |  | 8/8 | 0·93(0·23-3·82) |  | 29/13 | 2·16(0·67-6·98) | 0·1353 |
|  |  |  |  |  |  |  |  |  |  |  |  |  |
| No alcohol | 8/14 | 1·00 (ref) |  | 15/16 | 1·82(0·47-7·06) |  | 9/20 | 0·93(0·21-4·05) |  | 27/17 | 3·89(1·07-14·12 | 0·0463 |
| Alcohol < median (20 g/day) | 19/21 | 1·00 (ref) |  | 11/23 | 0·54(0·19-1·56) |  | 16/23 | 0·95(0·33-2·72) |  | 45/21 | 3·3(1·24-8·81) | 0·0034 |
| Alcohol ≥ median | 17/29 | 1·00 (ref) |  | 10/23 | 0·69(0·2-2·37) |  | 10/20 | 0·37(0·1-1·38) |  | 17/24 | 0·96(0·31-2·96) | 0·8011 |
|  |  |  |  |  |  |  |  |  |  |  |  |  |
| **5-MTHF** |  |  |  |  |  |  |  |  |  |  |  |  |
| Males | 48/52 | 1·00 (ref) |  | 52/53 | 1·28 (0·68-2·43) |  | 36/51 | 0·92 (0·46-1·84) |  | 35/52 | 0·88 (0·45-1·75) | 0·53 |
|  |  |  |  |  |  |  |  |  |  |  |  |  |
| <65 years | 32/31 | 1·00 (ref) |  | 27/33 | 0·73 (0·32-1·65) |  | 14/33 | 0·45 (0·18-1·17) |  | 22/28 | 0·91 (0·38-2·16) | 0·57 |
| ≥ 65 years | 29/31 | 1·00 (ref) |  | 33/30 | 1·52 (0·62-3·69) |  | 27/28 | 0·96 (0·38-2·43) |  | 19/34 | 0·76 (0·30-1·89) | 0·38 |
|  |  |  |  |  |  |  |  |  |  |  |  |  |
| BMI <25 | 11/16 | 1·00 (ref) |  | 12/23 | 0·90 (0·25-3·23) |  | 10/19 | 0·75 (0·19-2·96) |  | 14/19 | 1·36 (0·39-4·72) | 0·64 |
| BMI 25 to <30 | 26/35 | 1·00 (ref) |  | 25/30 | 1·50 (0·60-3·76) |  | 20/28 | 1·31 (0·49-3·51) |  | 22/28 | 1·31 (0·49-3·51) | 0·66 |
| BMI ≥ 30 | 23/11 | 1·00 (ref) |  | 23/9 | 1·03 (0·31-3·46) |  | 11/14 | 0·45 (0·12-1·64) |  | 5/14 | 0·14 (0·03-0·65) | 0·008 |
|  |  |  |  |  |  |  |  |  |  |  |  |  |
| *H· pylori* - | 32/26 | 1·00 (ref) |  | 30/24 | 0·95 (0·38-2·34) |  | 14/24 | 0·35 (0·12-1·01) |  | 23/24 | 0·96 (0·38-2·4) | 0·54 |
| *H· pylori* + | 27/35 | 1·00 (ref) |  | 24/37 | 1·06 (0·48-2·36) |  | 25/36 | 1·04 (0·45-2·4) |  | 15/35 | 0·62 (0·25-1·5) | 0·35 |
|  |  |  |  |  |  |  |  |  |  |  |  |  |
| Reflux - | 30/50 | 1·00 (ref) |  | 34/46 | 1·48 (0·72-3·06) |  | 21/51 | 0·83 (0·37-1·82) |  | 20/23 | 0·86 (0·39-1·87) | 0·41 |
| Reflux + | 31/12 | 1·00 (ref) |  | 26/16 | 0·45 (0·15-1·29) |  | 20/10 | 0·53 (0·16-1·74) |  | 21/9 | 0·88 (0·28-2·79) | 0·91 |
|  |  |  |  |  |  |  |  |  |  |  |  |  |
| Never smoker | 9/28 | 1·00 (ref) |  | 13/18 | 2·94(0·88-9·8) |  | 9/24 | 1·95(0·56-6·83) |  | 9/26 | 1·42(0·43-4·68) | 0·6908 |
| Former smoker | 28/15 | 1·00 (ref) |  | 28/31 | 0·7(0·27-1·82) |  | 17/27 | 0·49(0·17-1·42) |  | 18/31 | 0·35(0·12-1·00) | 0·0387 |
| Current smoker | 23/17 | 1·00 (ref) |  | 19/11 | 0·92(0·30-2·84) |  | 12/9 | 0·71(0·20-2·49) |  | 14/5 | 1·46(0·37-5·80) | 0·8219 |
|  |  |  |  |  |  |  |  |  |  |  |  |  |
| No alcohol | 20/11 | 1·00 (ref) |  | 16/21 | 0·48(0·14-1·59) |  | 11/16 | 0·16(0·04-0·69) |  | 12/18 | 0·65(0·19-2·27) | 0·3066 |
| Alcohol < median (20 g/day) | 24/26 | 1·00 (ref) |  | 31/22 | 1·47(0·6-3·61) |  | 18/18 | 1·18(0·45-3·10) |  | 17/21 | 0·75(0·28-2·00) | 0·5434 |
| Alcohol ≥ median | 17/25 | 1·00 (ref) |  | 13/20 | 1·28(0·39-4·23) |  | 12/27 | 1·61(0·48-5·44) |  | 12/23 | 1·59(0·49-5·14) | 0·4053 |
|  |  |  |  |  |  |  |  |  |  |  |  |  |
| **PLP** |  |  |  |  |  |  |  |  |  |  |  |  |
| Males | 115/53 | 1·00 (ref) |  | 28/53 | 0·28 (0·15-0·53) |  | 17/53 | 0·21 (0·10-0·43) |  | 11/52 | 0·20 (0·09-0·45) | <0·0001 |
|  |  |  |  |  |  |  |  |  |  |  |  |  |
| <65 years | 59/22 | 1·00 (ref) |  | 14/33 | 0·16 (0·07-0·39) |  | 14/36 | 0·20 (0·08-0·49) |  | 8/36 | 0·13 (0·04-0·38) | <0·0001 |
| ≥ 65 years | 81/41 | 1·00 (ref) |  | 17/30 | 0·22 (0·09-0·54) |  | 3/27 | 0·05 (0·01-0·26) |  | 6/26 | 0·24 (0·08-0·71) | <0·0001 |
|  |  |  |  |  |  |  |  |  |  |  |  |  |
| BMI <25 | 30/19 | 1·00 (ref) |  | 6/19 | 0·23 (0·06-0·84) |  | 4/20 | 0·26 (0·07-1·00) |  | 7/20 | 0·33 (0·09-1·20) | 0·04 |
| BMI 25 to <30 | 33/8 | 1·00 (ref) |  | 12/21 | 0·27 (0·11-0·66) |  | 7/12 | 0·16 (0·05-0·51) |  | 10/8 | 0·21 (0·07-0·65) | 0·0004 |
| BMI ≥ 30 | 47/10 | 1·00 (ref) |  | 8/14 | 0·05 (0·01-0·24) |  | 6/12 | 0·10 (0·02-0·43) |  | 1/13 | 0·01 (0·00-0·16) | <0·0001 |
|  |  |  |  |  |  |  |  |  |  |  |  |  |
| *H· pylori* - | 67/25 | 1·00 (ref) |  | 20/27 | 0·33 (0·15-0·77) |  | 5/29 | 0·12 (0·04-0·39) |  | 6/19 | 0·25 (0·08-0·78) | 0·0005 |
| *H· pylori* + | 66/38 | 1·00 (ref) |  | 9/33 | 0·12 (0·05-0·33) |  | 9/34 | 0·17 (0·07-0·43) |  | 7/39 | 0·14 (0·05-0·39) | <0·0001 |
|  |  |  |  |  |  |  |  |  |  |  |  |  |
| Reflux - | 69/46 | 1·00 (ref) |  | 16/47 | 0·25 (0·11-0·53) |  | 10/54 | 0·13 (0·05-0·32) |  | 9/54 | 0·17 (0·07-0·41) | <0·0001 |
| Reflux + | 71/17 | 1·00 (ref) |  | 15/16 | 0·25 (0·09-0·67) |  | 7/8 | 0·25 (0·07-0·89) |  | 5/8 | 0·21 (0·05-0·90) | 0·0015 |
|  |  |  |  |  |  |  |  |  |  |  |  |  |
| Never smoker | 32/23 | 1·00 (ref) |  | 4/22 | 0·12(0·03-0·45) |  | 2/26 | 0·04(0·01-0·22) |  | 1/26 | 0·02(0-0·21) | <·0001 |
| Former smoker | 60/26 | 1·00 (ref) |  | 16/23 | 0·21(0·08-0·58) |  | 8/26 | 0·17(0·06-0·52) |  | 7/30 | 0·21(0·07-0·61) | 0·0003 |
| Current smoker | 45/13 | 1·00 (ref) |  | 10/15 | 0·15(0·04-0·54) |  | 7/10 | 0·22(0·05-0·87) |  | 6/5 | 0·17(0·03-0·96) | 0·0128 |
|  |  |  |  |  |  |  |  |  |  |  |  |  |
| No alcohol | 39/26 | 1·00 (ref) |  | 10/17 | 0·52(0·16-1·64) |  | 5/8 | 0·46(0·11-1·9) |  | 4/16 | 0·34(0·08-1·42) | 0·0775 |
| Alcohol < median (20 g/day) | 68/24 | 1·00 (ref) |  | 11/20 | 0·2(0·08-0·53) |  | 5/26 | 0·05(0·01-0·21) |  | 6/19 | 0·14(0·05-0·46) | <·0001 |
| Alcohol ≥ median | 33/13 | 1·00 (ref) |  | 10/26 | 0·13(0·04-0·44) |  | 7/29 | 0·16(0·05-0·56) |  | 4/27 | 0·15(0·03-0·68) | 0·0030 |

Adjusted for sex (male/female except stratified by sex), age (years), smoking status (current/previous/never), BMI 5 years prior, location (Northern Ireland/Republic of Ireland), education (years), occupation (manual/non-manual), alcohol intake (none, <median, ≥median), *Helicobacter pylori* infection (seropositive/seronegative), gastroesophageal reflux symptoms (ever/never), regular NSAID use (ever/never) unless used as the stratification variable.
